# Supplementary material for: Modeling glioblastoma heterogeneity as a dynamic network of cell states
Source: Mol Syst Biol. 2021 Sep 16;17(9):e10105. doi: 10.15252/msb.202010105 (PMC8444284; doi:10.15252/msb.202010105)
Supplement: Supplementary file 5 — Source Data for Figure 3 [file MSB-17-e10105-s001.zip › Figure3A_sourcedata/GSEA_3065/hallmarks_state5.GseaPreranked.1623416302988/HALLMARK_PI3K_AKT_MTOR_SIGNALING.html]

Details for gene set HALLMARK\_PI3K\_AKT\_MTOR\_SIGNALING[GSEA]

|  || Dataset | state5 |
| Phenotype | NoPhenotypeAvailable |
| Upregulated in class | na\_neg |
| GeneSet | HALLMARK\_PI3K\_AKT\_MTOR\_SIGNALING |
| Enrichment Score (ES) | -0.4972078 |
| Normalized Enrichment Score (NES) | -1.6664324 |
| Nominal p-value | 0.0 |
| FDR q-value | 0.011890259 |
| FWER p-Value | 0.073 |
Table: GSEA Results Summary

  

Fig 1: Enrichment plot: HALLMARK\_PI3K\_AKT\_MTOR\_SIGNALING      
 Profile of the Running ES Score & Positions of GeneSet Members on the Rank Ordered List

  

| PROBE | GENE SYMBOL | GENE\_TITLE | RANK IN GENE LIST | RANK METRIC SCORE | RUNNING ES | CORE ENRICHMENT || 1 | HSP90B1 |  |  | 33 | 0.729 | 0.0386 | No |
| 2 | CALR |  |  | 55 | 0.631 | 0.0728 | No |
| 3 | MAPK10 |  |  | 376 | 0.350 | 0.0572 | No |
| 4 | CXCR4 |  |  | 450 | 0.325 | 0.0678 | No |
| 5 | TSC2 |  |  | 703 | 0.261 | 0.0547 | No |
| 6 | SLC2A1 |  |  | 1190 | 0.186 | 0.0109 | No |
| 7 | RAF1 |  |  | 1275 | 0.175 | 0.0116 | No |
| 8 | STAT2 |  |  | 1350 | 0.166 | 0.0129 | No |
| 9 | TNFRSF1A |  |  | 1377 | 0.163 | 0.0194 | No |
| 10 | SMAD2 |  |  | 1426 | 0.159 | 0.0233 | No |
| 11 | EGFR |  |  | 1547 | 0.147 | 0.0183 | No |
| 12 | PIK3R3 |  |  | 1584 | 0.142 | 0.0225 | No |
| 13 | PTEN |  |  | 2191 | 0.099 | -0.0398 | No |
| 14 | NCK1 |  |  | 2405 | 0.085 | -0.0588 | No |
| 15 | PLCG1 |  |  | 2435 | 0.083 | -0.0572 | No |
| 16 | PIKFYVE |  |  | 2596 | 0.074 | -0.0709 | No |
| 17 | TBK1 |  |  | 2615 | 0.073 | -0.0687 | No |
| 18 | ADCY2 |  |  | 2745 | 0.066 | -0.0794 | No |
| 19 | ITPR2 |  |  | 2803 | 0.063 | -0.0822 | No |
| 20 | PRKAA2 |  |  | 3146 | 0.044 | -0.1180 | No |
| 21 | VAV3 |  |  | 3333 | 0.035 | -0.1369 | No |
| 22 | PRKAG1 |  |  | 3484 | 0.028 | -0.1522 | No |
| 23 | CLTC |  |  | 3513 | 0.027 | -0.1538 | No |
| 24 | TIAM1 |  |  | 3782 | 0.016 | -0.1829 | No |
| 25 | MAP3K7 |  |  | 3835 | 0.014 | -0.1880 | No |
| 26 | MKNK1 |  |  | 3851 | 0.014 | -0.1889 | No |
| 27 | ACACA |  |  | 3928 | 0.010 | -0.1968 | No |
| 28 | RPS6KA3 |  |  | 4037 | 0.006 | -0.2086 | No |
| 29 | MKNK2 |  |  | 4083 | 0.004 | -0.2134 | No |
| 30 | MAP2K6 |  |  | 4120 | 0.003 | -0.2173 | No |
| 31 | PPP2R1B |  |  | 4307 | -0.006 | -0.2379 | No |
| 32 | TRAF2 |  |  | 4326 | -0.007 | -0.2395 | No |
| 33 | PLA2G12A |  |  | 4345 | -0.008 | -0.2411 | No |
| 34 | NFKBIB |  |  | 4459 | -0.013 | -0.2530 | No |
| 35 | CDK2 |  |  | 4489 | -0.013 | -0.2555 | No |
| 36 | PAK4 |  |  | 4492 | -0.014 | -0.2550 | No |
| 37 | IRAK4 |  |  | 4721 | -0.023 | -0.2792 | No |
| 38 | CDK1 |  |  | 4766 | -0.025 | -0.2828 | No |
| 39 | MAPK8 |  |  | 4964 | -0.032 | -0.3031 | No |
| 40 | DDIT3 |  |  | 5335 | -0.046 | -0.3420 | No |
| 41 | MAPK1 |  |  | 5469 | -0.051 | -0.3539 | No |
| 42 | ATF1 |  |  | 5691 | -0.062 | -0.3752 | No |
| 43 | GRK2 |  |  | 5736 | -0.063 | -0.3764 | No |
| 44 | RIT1 |  |  | 5824 | -0.066 | -0.3824 | No |
| 45 | RPS6KA1 |  |  | 5842 | -0.067 | -0.3804 | No |
| 46 | CAB39 |  |  | 5893 | -0.069 | -0.3820 | No |
| 47 | MAP2K3 |  |  | 5956 | -0.072 | -0.3848 | No |
| 48 | CDKN1B |  |  | 5996 | -0.074 | -0.3849 | No |
| 49 | RIPK1 |  |  | 6162 | -0.083 | -0.3986 | No |
| 50 | MAPK9 |  |  | 6194 | -0.085 | -0.3972 | No |
| 51 | DUSP3 |  |  | 6214 | -0.086 | -0.3943 | No |
| 52 | E2F1 |  |  | 6528 | -0.103 | -0.4235 | No |
| 53 | MYD88 |  |  | 6788 | -0.120 | -0.4457 | No |
| 54 | AKT1S1 |  |  | 6810 | -0.122 | -0.4410 | No |
| 55 | ECSIT |  |  | 6815 | -0.122 | -0.4343 | No |
| 56 | GSK3B |  |  | 6951 | -0.133 | -0.4418 | No |
| 57 | PRKAR2A |  |  | 7042 | -0.141 | -0.4438 | No |
| 58 | RALB |  |  | 7093 | -0.145 | -0.4410 | No |
| 59 | EIF4E |  |  | 7292 | -0.166 | -0.4536 | No |
| 60 | GRB2 |  |  | 7531 | -0.195 | -0.4690 | No |
| 61 | ACTR2 |  |  | 7783 | -0.231 | -0.4838 | Yes |
| 62 | AKT1 |  |  | 7805 | -0.235 | -0.4726 | Yes |
| 63 | MAPKAP1 |  |  | 7898 | -0.255 | -0.4681 | Yes |
| 64 | ACTR3 |  |  | 8011 | -0.277 | -0.4646 | Yes |
| 65 | SQSTM1 |  |  | 8044 | -0.284 | -0.4517 | Yes |
| 66 | PTPN11 |  |  | 8160 | -0.312 | -0.4466 | Yes |
| 67 | UBE2D3 |  |  | 8163 | -0.314 | -0.4286 | Yes |
| 68 | PPP1CA |  |  | 8193 | -0.324 | -0.4131 | Yes |
| 69 | CSNK2B |  |  | 8298 | -0.354 | -0.4042 | Yes |
| 70 | UBE2N |  |  | 8529 | -0.447 | -0.4042 | Yes |
| 71 | ARPC3 |  |  | 8591 | -0.482 | -0.3831 | Yes |
| 72 | ARF1 |  |  | 8606 | -0.495 | -0.3560 | Yes |
| 73 | PIN1 |  |  | 8623 | -0.509 | -0.3283 | Yes |
| 74 | CDK4 |  |  | 8625 | -0.510 | -0.2988 | Yes |
| 75 | HRAS |  |  | 8633 | -0.514 | -0.2698 | Yes |
| 76 | ARHGDIA |  |  | 8659 | -0.531 | -0.2418 | Yes |
| 77 | CDKN1A |  |  | 8679 | -0.545 | -0.2124 | Yes |
| 78 | AP2M1 |  |  | 8743 | -0.600 | -0.1847 | Yes |
| 79 | YWHAB |  |  | 8855 | -0.715 | -0.1557 | Yes |
| 80 | RAC1 |  |  | 8919 | -0.815 | -0.1156 | Yes |
| 81 | PFN1 |  |  | 8977 | -1.045 | -0.0614 | Yes |
| 82 | CFL1 |  |  | 8978 | -1.071 | 0.0007 | Yes |
Table: GSEA details [plain text format]

  

Fig 2: HALLMARK\_PI3K\_AKT\_MTOR\_SIGNALING: Random ES distribution      
 Gene set null distribution of ES for **HALLMARK\_PI3K\_AKT\_MTOR\_SIGNALING**
